# Worldwide Research Trends on Medicinal Plants

**DOI:** 10.3390/ijerph17103376

**Published:** 2020-05-12

**Authors:** Esther Salmerón-Manzano, Jose Antonio Garrido-Cardenas, Francisco Manzano-Agugliaro

**Affiliations:** 1 Faculty of Law, Universidad Internacional de La Rioja (UNIR), 26006 Logroño, Spain; esther.salmeron@unir.net; 2 Department of Biology and Geology, University of Almeria, ceiA3, 04120 Almeria, Spain; jcardena@ual.es; 3 Department of Engineering, University of Almeria, ceiA3, 04120 Almeria, Spain

**Keywords:** medicinal plants, drugs, worldwide research, bibliometrics, traditional medicine

## Abstract

The use of medicinal plants has been done since ancient times and may even be considered the origin of modern medicine. Compounds of plant origin have been and still are an important source of compounds for drugs. In this study a bibliometric study of all the works indexed in the Scopus database until 2019 has been carried out, analyzing more than 100,000 publications. On the one hand, the main countries, institutions and authors researching this topic have been identified, as well as their evolution over time. On the other hand, the links between the authors, the countries and the topics under research have been analyzed through the detection of communities. The last two periods, from 2009 to 2014 and from 2015 to 2019, have been examined in terms of research topics. It has been observed that the areas of study or clusters have been reduced, those of the last period being those engaged in unclassified drug, traditional medicine, cancer, in vivo study—antidiabetic activity, and animals—anti-inflammatory activity. In summary, it has been observed that the trend in global research is focused more on the search for new medicines or active compounds rather than on the cultivation or domestication of plant species with this demonstrated potential.

## 1. Introduction

Ten percent of all vascular plants are used as medicinal plants [1], and there are estimated to be between 350,000 [2] and almost half a million [3] species of them. Since ancient times, plants have been used in medicine and are still used today [4]. In the beginning, the trial and error method was used to treat illnesses or even simply to feel better, and in this way, to distinguish useful plants with beneficial effects [5]. The use of these plants has been gradually refined over the generations, and this has become known in many contexts as traditional medicine. The official definition of traditional medicine can be considered as “the sum total of the knowledge, skills and practices based on the theories, beliefs and experiences indigenous to different cultures, whether explicable or not, used in the maintenance of health, as well as in the prevention, diagnosis, improvement or treatment of physical and mental illnesses” [6].

It is a fact that all civilizations have developed this form of medicine [7] based on the plants in their own habitat [8]. There are even authors who claim that this transmitted knowledge is the origin of medicine and pharmacy. Even today, hundreds of higher plants are cultivated worldwide to obtain useful substances in medicine and pharmacy [9]. The therapeutic properties of plants gave rise to medicinal drugs made from certain plants with these benefits [10].

Until the 18th century, the therapeutic properties of many plants, their effect on the human organism and their method of treatment were known, but the active compound was unknown [11]. As an example, the Canon of Medicine written by the Persian physician and scientist Avicenna (Ibn Sina) was used until the 18th century [12].

The origin of modern science, especially in the Renaissance, in particular chemical analysis, and the associated instrumentation such as the microscope, was what made it possible to isolate the active principles of medical plants [13]. Since then, these active principles have been obtained synthetically in the laboratory to produce the medicines later [14]. The use of medicines was gradually expanded. Until today, the direct use of medicinal plants is apparently displaced in modern medicine [15]. Today’s medicine needs the industry producing pharmaceutical medicines, which are largely based on the active principles of plants, and therefore, these are used as raw materials in many cases [16]. Yet, today, the underdeveloped world does not have access to this modern medicine of synthetic origin, and therefore, large areas of the world continue to use traditional medicine based on the direct use of medicinal plants due to their low cost [17].

However, it should be noted that the possible trend to return to this type of traditional medicine may have two major drawbacks. The first is the use of medicinal plants without sanitary control, without thinking about the possible harmful aspects for health [18]. Although many plants do not have side effects like the aromatic plants used in infusions: chamomile, rosemary, mint, or thyme; however, others may have dangerous active principles. To cite an example, Bitter melon (*Momordica charantia L.*) used to cure fever and in cases of malaria [19], its green seeds are very toxic as they can cause a sharp drop in blood sugar and induce a patient’s coma (hypoglycemic coma) [20]; this is due to the fact that the components of bitter melon extract appear to have structural similarities to animal insulin [21]. Secondly, there has been a proliferation of products giving rise to false perspectives, as they are not sufficiently researched [22].

Examining the specialized literature of reviews and bibliometric studies on medicinal plants, three types of studies are found: those focused on a geographical area, those focused on a specific plant or family, and those focused on some type of medical interest activity. Regarding the studies of geographical areas, for example, there are the studies of Africa. Specifically, in South Africa, the plants that are marketed [23], as these plants of medical interest have been promoted [24], or for the treatment of specific diseases such as Alzheimer’s [25]. In Central Africa, the studies of Cameroon are remarkable, where for general bibliometric studies of its scientific output, the topic of medicinal plants stands out as one of the most important in this country [26]. Or those of Ghana, regarding frequent diseases in this country such as malaria, HIV/AIDS, hypertension, tuberculosis, or bleeding disorders [27]. Other countries that have conducted a bibliometric study of their medicinal plants have been Cuba [28] and China [29].

The other direction of the bibliometric studies mentioned, those that focus on specific plants, are those of: *Artemisia annua L.* [30], *Aloe vera* [31], *Panax ginseng* [32], *Punica grantum* L. [33], *Apocynum cannabinum* [34], or *Andrographis paniculata* [35]. The third line of the bibliometric research on medicinal plants deals with some kind of specific activity; there are studies for example for the activities of: antibacterial or antifungal [36], antioxidant [37], and anticancer [38,39,40].

As a common feature of the bibliometric studies published so far, none of them has a worldwide perspective. Furthermore, they are generally based on Web of Science and some of them on other more specific databases such as CAB Abstracts or PlantMedCUBA, but no work based on Scopus has been observed. Therefore, this paper aims to study what types of scientific advances are being developed around medicinal plants, what research trends are being carried out, and by which countries and research institutions. To this purpose, it is proposed to carry out a bibliometric analysis of all the scientific publications on this topic.

## 2. Materials and Methods

**Figure 1:**
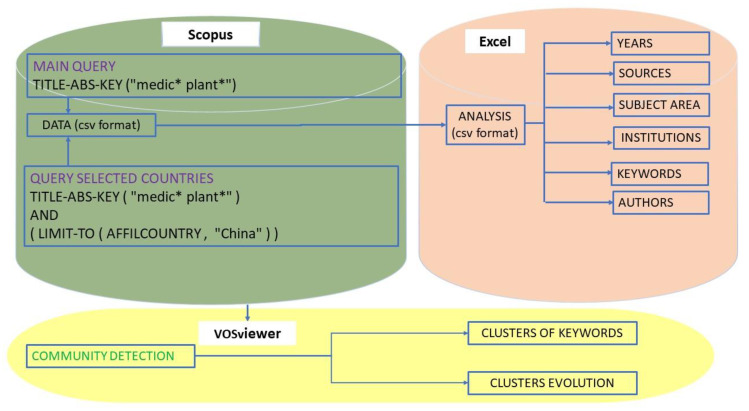
Methodology.

The data analyzed in this work have been obtained through a query in the Scopus database, which has been successfully used in a large number of bibliometric studies [41]. Due to the large amount of results, it was necessary to use the Scopus API to download the data, whose methodology has been developed in previous works [42,43]. In this study, the query used was: (TITLE-ABS-KEY(“medic* plant*”)). An outline of the methodology used is shown in Figure 1. The analysis of the scientific communities, both in terms of keywords and the relationship between authors or between countries was done with the SW VosViewer [44].

## 3. Results

### 3.1. Global Evolution Trend

**Figure 2:**
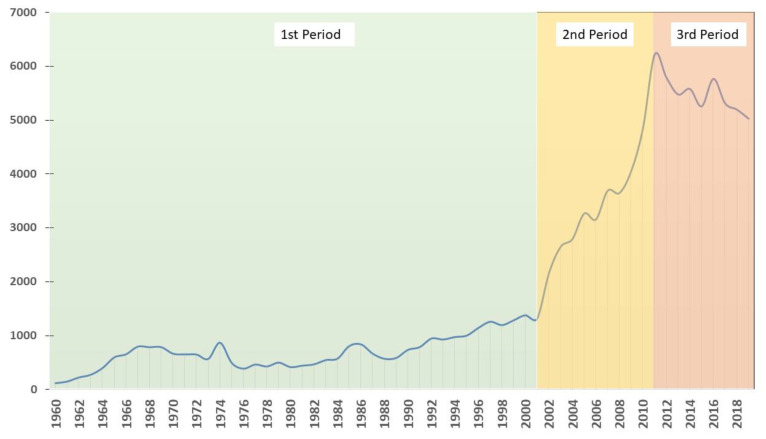
Worldwide temporal evolution of medical plants publications.

From 1960 to 2019, more than 110,000 studies related to medicinal plants have been published. Figure 2 shows the trend in research in this field. Overall, it can be said that there was a continuous increase from 1960 to 2001, with just over 1300 published studies. From here, the trend increases faster until 2011, when it reaches a maximum of just over 6200 publications. After this period, publications stabilize at just over 5000 per year. These three periods identified are highlighted in Figure 2.

### 3.2. Global Subject Category

**Figure 3:**
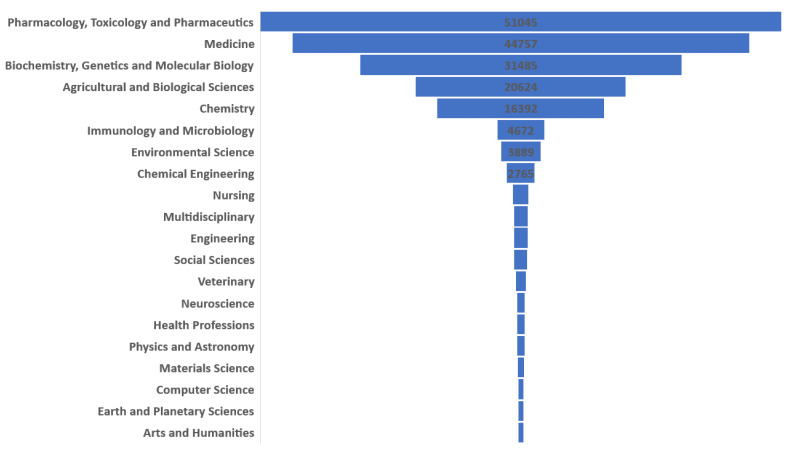
Medicinal plants publications by scientific categories indexed in Scopus.

If the results are analyzed according to the categories in which they have been published (see Figure 3), according to the Scopus database, it can be seen that most of them have been carried out in the Pharmacology, Toxicology and Pharmaceutics category with 27.1 % of the total. Other categories with significant relative relevance have been: Medicine (23.8%), Biochemistry, Genetics and Molecular Biology (16.7%), Agricultural and Biological Sciences (11%), Chemistry (8.7%), Immunology and Microbiology (2.5%), Environmental Science (2.1%), and Chemical Engineering (1.5%). All other categories are below 1%, such as: Nursing, Multidisciplinary, or Engineering.

### 3.3. Distribution of Publications by Countries

**Figure 4:**
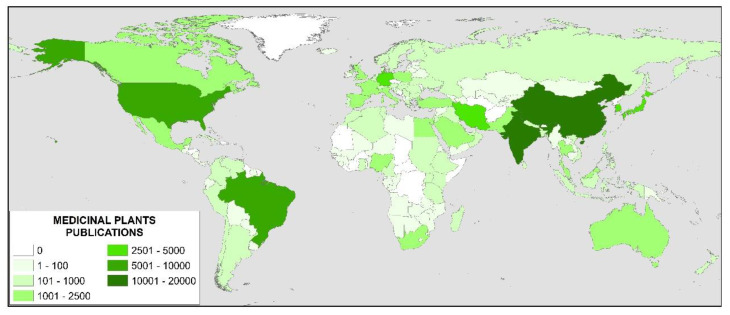
Worldwide research on medical plants.

If the results obtained are analyzed by country, a total of 159 countries have published on this topic. Figure 4 shows the countries that have published on the subject and the intensity with which they published has been shown. It is observed that China and India stand out over the rest of the countries with more than 10,000 publications, perhaps influenced by traditional medicine, although their most cited works are related to antioxidant activity, both for China [45], and for India [46,47], and in this last country also antidiabetic potential [4]. The third place is the USA followed by Brazil, both with more than 5000 publications. The most frequently cited publications from these countries focus on antioxidant activity [48], and antimicrobial activity [49] for the USA and anti-inflammatory activity for Brazil [50,51].

**Figure 5:**
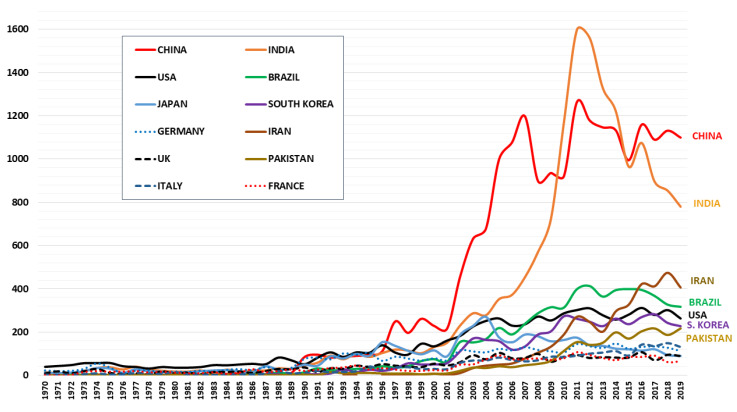
Temporal evolution on medical plants publications for Top 12 countries.

As mentioned, the list of countries is very long, but those with more than 2000 publications are included: Japan, South Korea, Germany, Iran, United Kingdom, Pakistan, Italy, and France. If the overall results obtained are analyzed in their evolution by years, for this list of countries with more than 2000 publications, Figure 5 is obtained. From this point onwards, three groups of countries can be identified.

The first group is the leaders of this research, China and India, with between 800 and 1100 publications per year. China led the research from 1996 to 2010, and from this year to 2016, the leader was India, after which it returned to China. The second group of five countries is formed in order in the last year of the study: Iran, Brazil, USA, South Korea and Pakistan. This group of countries has a sustained growth over time, with a rate of publications between 200 and 400 per year. It should be noted that Brazil led the third place for a decade, from 2007 to 2016, since then that position is for Iran. The third group of five countries is made up of: Japan, Germany, United Kingdom, Italy, and France. They are keeping the publications around 100 a year, with an upward trend, but at a very slight rate.

**Figure 6:**
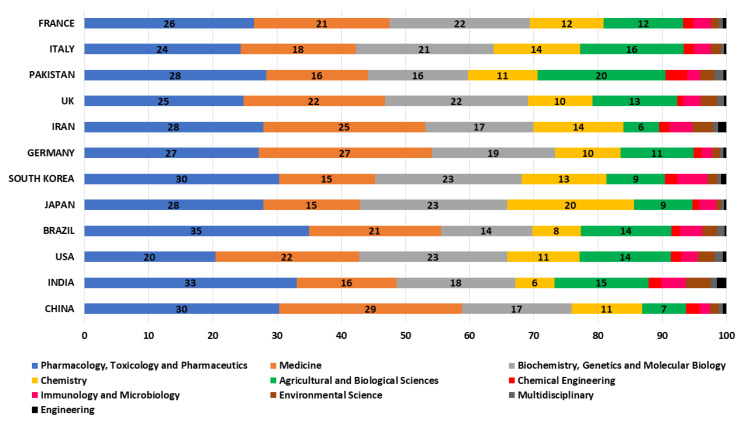
Distribution by scientific categories according to countries.

If the analysis of the publications by country is made according to the categories in which they publish, Figure 6 is obtained, which shows the relative effort between the different themes or categories is shown. At first look, it might seem that they have a similar distribution. However, in relative terms the category of Pharmacology, Toxicology and Pharmaceutics is led by Brazil with 35% of its own publications followed by India with 33%. For the Medicine category, in relative terms it is led by China with 29 %, followed by Germany with 27 %. The category of Biochemistry, Genetics and Molecular Biology always takes second or third place for this ranking of countries, standing out especially for Japan and South Korea with 23% and for France with 22%. The fourth category for many countries is Agricultural and Biological Sciences, with Pakistan standing out with 20%, followed by Italy with 16%. The category of Chemistry occupies the fourth category for countries such as Japan with 20% or Iran with 14%. The other categories: Chemical Engineering, Immunology and Microbiology, Environmental Science, Multidisciplinary, or Engineering, are below 5 % in all countries.

According to these results, it can be seen the relative lack of relevance of the category of Agricultural and Biological Sciences for medicinal plants, compared to the categories of Pharmacology, Toxicology and Pharmaceutics, Medicine, or Biochemistry, Genetics and Molecular Biology.

### 3.4. Institutions (Affiliations)

So far, the distribution by country has been seen, but the research is done in specific research centers (institution or affiliations as are indexed in Scopus) and therefore, it is important to study them. Table 1 shows the 25 institutions with more than 400 publications, of which 13 are from China (including the first 7), 3 from Brazil, 2 from South Korea, and now with 1: Saudi Arabia, Pakistan, Iran, Mexico, Cameroon, France, and Malaysia.

If the three main keywords of these affiliations are analyzed, it can be seen that there are no great differences, and in fact, they are often the same: Unclassified Drug, Drug Isolation, Drug Structure, Chemistry, Controlled Study, Isolation And Purification, Chemistry, and Plant Extract. They only call attention to “Drugs, Chinese Herbal” which appears in two affiliations: China Academy of Chinese Medical Sciences, and Beijing University of Chinese Medicine, which of course is a very specific issue in this country.

### 3.5. Authors

The main authors researching this topic are shown in Table 2, which are those with more than 100 publications on this topic. It is observed that they are authors with a significantly high h-index. On the other hand, it is noteworthy that the first two are not from China or India, which as we have seen were the most productive countries, and also had the most relevant institutions in this area. The lead author is from South Africa, J. Van Staden, and the second from Bangladesh, M. Rahmatullah. The author with the highest h-index is from Germany, T. Efferth.

**Figure 7:**
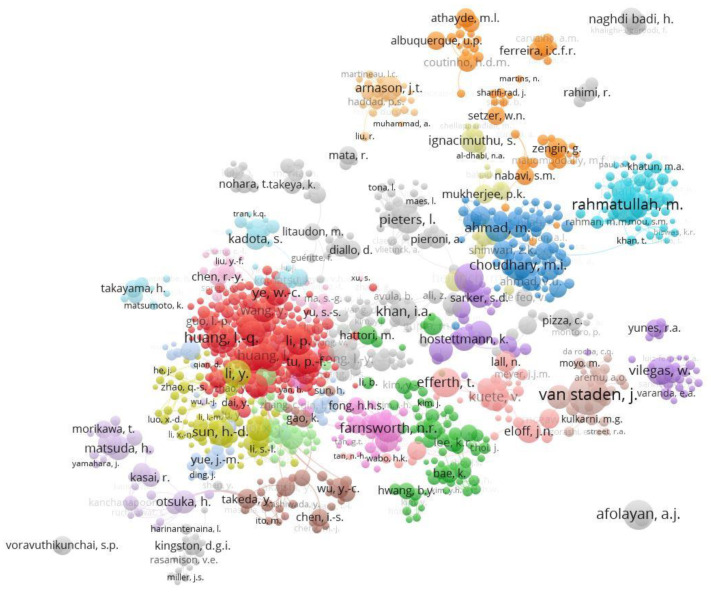
A collaborative network of authors with more than 40 publications on medicinal plants.

If the network of collaboration between authors with more than 40 documents is established, Figure 7 is obtained. Here, there are 33 clusters, where the most important is the red one with 195 authors, where the central author is Huang, L.Q. The second more abundant cluster is the green one, composed of 69 authors. In this cluster, there is no central author, but instead, a collaboration between prominent authors such as Kim, J.S., Lee, K.R. or Park, J.S. The third cluster, in blue, is composed of 64 authors, led by the authors M.I. Choudhary and M. Ahmad.

The fourth cluster, of yellow color is composed of 63 authors, the central authors are Y. Li and H-D. Sun. The fifth cluster, in purple, is also composed of 51 authors, the central author is W. Villegas. It should be noted that this cluster is not linked to the whole network, so they must research very specific topics in their field. The sixth cluster is composed of 48 authors and is cyan colored, the central author is Rahmatullah, M. The cluster of the main author of Table 2, Van Staden, J., is composed of 23 authors, and would be number 17 in order of importance by number of authors, is light brown, and is located next to that of W. Vilegas but without any apparent connection.

### 3.6. Keywords

#### 3.6.1. Global Perspective

**Figure 8:**
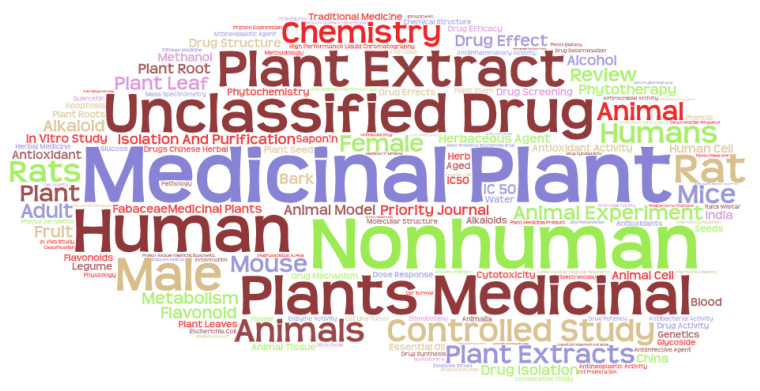
Cloudword of keywords in medical plants publications.

The central aspect of bibliometric studies is to study the keywords in the publications and, through the relationships between them, to establish the clusters or scientific communities in which the different topics associated with a field of study can be grouped together. If keywords are extracted from the total number of publications, an overview can be made of the most used keywords in relation to the subject of medicinal plants (see Figure 8). As expected, the search terms are the main ones, but then, there are two indexing terms, Human and Nonhuman, and then Unclassified Drug and Plant Extract.

If the keywords are analyzed by country, and we do not take into account the search terms, the results are obtained in Table 3, where the four main keywords of the main countries that research this topic are shown. It can be seen that the terms: Unclassified Drug, Plant Extract, and Controlled Study, are the ones that dominate without a doubt.

#### 3.6.2. Keywords Related to Plants

If this keyword analysis is done by parts of the plant (see Table 4), which shows which parts of the plant have been most investigated. It should be noted that the number of documents is less than the sum of the individual keywords, since a publication contains more than one keyword. It has been obtained that the parts of the plant most studied in order of importance have been the value expressed in relative terms: Leaf-Leaves (33%), Root-Roots (22%), Seed (12%), Stem (10%), Fruit (10%), Bark (7%), and Flower (6%). The table also shows which plant families have been most used for the study of that part of the plant.

To give an idea of the most studied plant families, see Table 5. Although the first two are the same family, it has been left separately to indicate the indexing preferences of the two main affiliations that study them. This is also the situation with Compositae that correspond to the family of Asteraceae. This table lists for each plant family the main institution working on its study. However, it is curious that even if a country is a leader in certain studies related to plant families, most often it is found that the institution leading the issue is not from the country leading the study on that plant family. This helps to establish a certain amount of global leadership on the side of the institutions.

### 3.7. Clusters

**Figure 9:**
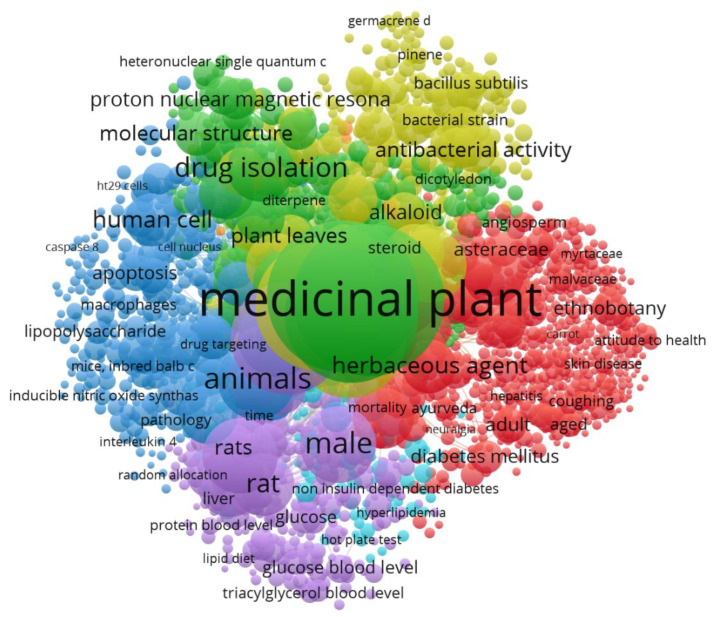
Network of keywords in medical plants publications: Clusters between 2009–2014.

The analysis of the clusters formed by the keywords allows the classification of the different groups into which the research trends are grouped. A first analysis has been made with the documents published between 2009 and 2019 and in two periods, from 2009 to 2014 and from 2015 to 2019. Figure 9 shows the clusters obtained for the period 2009 to 2014, showing seven clusters, which can be distinguished by color, and in Table 6 its main keywords have been collected.

The first of these clusters, in red (1-1), is linked to traditional medicine. This is reflected in the main keywords associated with this cluster: phytotherapy, herbaceous agent, traditional medicine, ethnobotany. Within this cluster, the most cited publications are related to the antioxidant function of plants. This includes the prevention of hyperglycemia hypertension [52], and the prevention of cancer. Of the latter, studies suggest that a reduced risk of cancer is associated with high consumption of vegetables and fruits [53]. Another topic frequently addressed is the antidiabetic properties, as some plants have hypoglycemic properties [34]. It should be remembered that diabetes mellitus is one of the common metabolic disorders, acquiring around 2.8% of the world’s population and is expected to double by 2025 [54].

The second cluster, in green (1-2), appears to be the central cluster, and is related to drugs—chemistry. The main keywords are: drug isolation, drug structure, chemistry, drug determination, and molecular structure. Here, the most cited publications are the search for new drugs [55] or in natural antimicrobials for food preservation [56].

The third cluster, in purple (1-3), is focused on in vivo study through studies with laboratory animals, as shown by keywords such as mouse and mice. As it is known that in vivo drug trials are initiated in laboratory animals such as mice, in general studies focused on anti-inflammatory effect [57,58].

The fourth cluster, in yellow (1-4), is engaged in the search for drugs. The main keywords in this regard are unclassified drug and drug screening. Within this cluster, the studies of flavonoids stand out [59]. Flavonoids have been shown to be antioxidant, free radical scavenger, coronary heart disease prevention, hepatoprotective, anti-inflammatory and anticancer, while some flavonoids show possible antiviral activities [60].

The fifth cluster, in blue (1-5), is focused on the effectiveness of some drugs, and their experimentation on animals. Some of the most cited publications of this cluster over this period are those focused on genus *Scutellaria* [61], Epimedium (*Berberidaceae*) [62] and Vernonia (*Asteraceae*) [63].

The sixth cluster, in cyan (1-6), is aimed at the effect of extraction solvent/technique on the antioxidant activity. One of the most cited publications in this regard studies the effects on barks of *Azadirachta indica*, *Acacia nilotica*, *Eugenia jambolana*, *Terminalia arjuna*, leaves and roots of *Moringa oleifera*, fruit of *Ficus religiosa*, and leaves of *Aloe barbadensis* [64]. Regarding neuroprotection, some publications are the related to genus *Peucedanum* [65] or *Bacopa monnieri* [66]. This cluster is among the clusters of traditional medicine (1-1) and drug efficacy (1-5).

Finally, the seventh orange cluster (1-7) is of small relative importance within this cluster analysis and is focused on malaria. As it is known, malaria is one of the most lethal diseases in the world every year [67]. Malaria causes nearly half a million deaths and was estimated at over 200 million cases, 90 per cent of which occurred in African countries [68]. Of the *Plasmodium* species affecting humans, *Plasmodium falciparum* causes the most deaths, although *Plasmodium vivax* is the most widely spread except in sub-Saharan Africa [69]. On the other hand, this cluster cites *Plasmodium berghei*, which mainly affects mice, and is often used as a model for testing medicines or vaccines [70].

**Figure 10:**
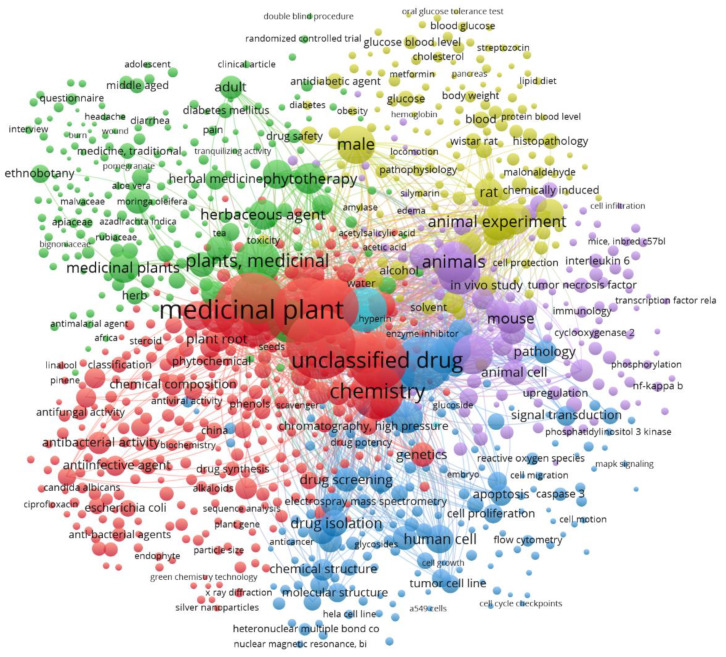
Network of keywords in medical plants publications: Clusters between 2015–2019.

The second period under study, from 2015 to 2019, is shown in Figure 10, where five clusters have been identified, Table 7, as opposed to the previous period which was seven. Now, there is no cluster focusing on malaria. In Figure 10, the colors of the cluster have been unified with those of Figure 9, when the clusters have the same topic as in the previous period.

The first cluster in order of importance (2-1), the red one in Figure 10, can be seen to be that of unclassified drug, which has gone from fourth place (1-4) to first in this last period. In this period, research works include one on the therapeutic potential of spirooxindoles as antiviral agents [71], or the antimicrobial peptides from plants [72].

The second cluster of this last period (2-2), the one in green in Figure 10, is the one assigned to traditional medicine, which has now moved up to second place (1-1) in decreasing order of significance. It seems that this cluster of traditional medicine is now the merging with the drug efficacy cluster of the previous period (1-4). This cluster includes research such as oxidative stress and Parkinson’s disease [73].

The cluster from the previous period that was devoted to animals-in vivo study (1-3), we assume is now divided into three new clusters. The first of these would be the third cluster (2-3), blue in Figure 10, which can be considered to be dedicated to cancer. One of the works in this cluster is “Anticancer activity of silver nanoparticles from Panax ginseng fresh leaves in human cancer cells” [74]. Then, the other two are committed to in vivo studies or with animals. The first one seems to be more engaged in vivo study at antidiabetic activity [75,76], would be the cyan-colored cluster 4 (2-4). The other cluster (2-5) involved in testing anti-inflammatory activity, with plants such as Curcumin [77], *Rosmarinus officinalis* [78], would be the purple cluster in Figure 10.

### 3.8. Collaboration Network of Countries

**Figure 11:**
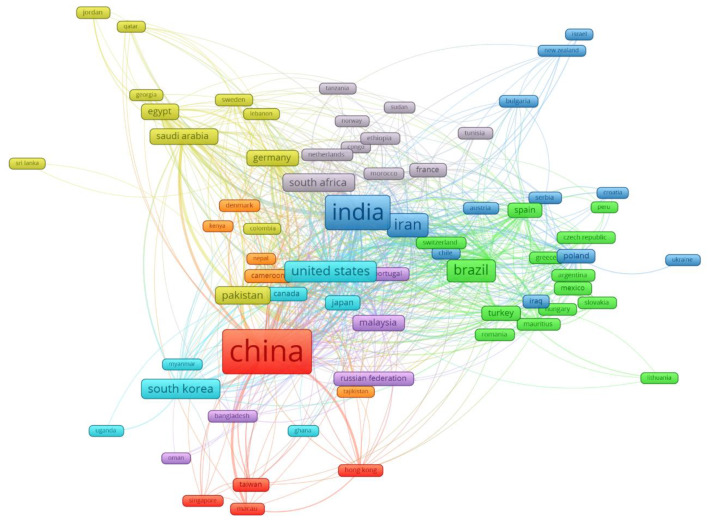
Countries network collaboration.

Figure 11 shows the collaborative network between countries doing research on medicinal plants. Table 8 lists the countries of each cluster identified and the main country of each cluster. The countries that are most central to this network of collaboration between countries are India, Iran, Indonesia, and the USA. The largest cluster is led by Brazil, which is also not restricted to its own geographical area as it has strong collaborative links with European countries as well as with neighboring countries such as Argentina. The second cluster led by South Africa also presents the same features as the previous one, some collaborations with nearby countries, Tanzania, Congo, or Sudan, but also with European countries such as France, Belgium, or the Netherlands.

The third cluster is led by India and has very strong collaboration with Iran, but it could also be considered as the central country in the whole international collaboration network. The cooperation with European countries comprises mainly Eastern countries like Poland, Serbia, or Croatia.

The fourth cluster, led by Germany and Pakistan, includes Middle Eastern countries such as Jordan, Saudi Arabia, and United Arab Emirates, which are quite related to the cluster led by China. The fifth cluster seems to have a geographical consideration within Asia by including countries such as Indonesia, Malaysia, Thailand, and Australia. The sixth cluster includes very technologically advanced countries such as USA, UK, Japan, Canada, or South Korea. The seventh cluster is very small in the number of countries. It is made up of very different countries like some in Africa: Cameroon and Kenya; some of Europe as Denmark, and some from Asia like Nepal. In this sense, most of the research linked to African countries in general and to Cameroon particularly is linked to the most frequent parasitic diseases [79], such as African trypanosomiasis [80], diarrhea [81] or tuberculosis [82]. Finally, the China cluster is made up of nearby areas of influence such as Taiwan, Singapore, Hong Kong, Macau, or Taiwan.

## 4. Conclusions

The use of plants as a source of research in the search for active compounds for medicine has been proven to have a significant scientific output. An analysis of the scientific literature indexed in the Scopus database concerning medicinal plants clearly shows that in the last 20 years, progress has been rapid, with a peak in 2010. From this year onwards, publications have stabilized at just over 5000 per year.

The research of products derived from the plants shows great collaboration between the countries of the first world and the countries with a traditional use of these plants from Asia, Africa or Latin America, all this to produce new medicines with scientific tests of safety and effectiveness. Within the analysis of the different clusters of collaboration between countries, there are four from Asia, led by China, India, Indonesia and Pakistan; two from Africa, led by South Africa and Cameroon, and then one from Latin America, led by Brazil and another from North America, led by the USA. It has been proven that there is no cluster of European countries, but that they generally collaborate with countries with which they have a commercial relationship. The research of medicinal plants in Africa is greatly underdeveloped, in contrast with China and India. In fact, there is no African country among the countries that published the most in this field. Among the first 25 institutions there is only one that belongs to the African continent. From this top 25, 13 are from China (including the first 7), 3 from Brazil, 2 from South Korea, and 1 of Saudi Arabia, Pakistan, Iran, Mexico, Cameroon, France, and Malaysia.

The most widely used search terms by the main institutions researching in this field are Unclassified Drug, Plant Extract, and Controlled Study. From the study of the keywords in the period from 2009 to 2014, seven clusters have been found, those dedicated to: Traditional medicine, Drug determination, Animals-in vivo study, Unclassified drug, Drug efficacy, Effect of extraction solvent, and Malaria. Subsequently, from the period 2015 to 2019, the clusters are reduced to five, and those focused on: Unclassified drug, Traditional medicine, Cancer, In vivo study—antidiabetic activity, and Animals—anti-inflammatory activity.

This is proven by the fact that of the total number of publications analyzed, more than 100,000, only 11% are in the Agricultural and Biological Sciences category, while more than 50% are grouped in the Pharmacology, Toxicology and Pharmaceutics category and Medicine. This study highlights the scarce research from the agronomic perspective regarding domestication, production or genetic or biotechnological research on breeding of medicinal plants.

## Figures and Tables

**Table 1:** Top 25 affiliations and main keywords.

Institution	Country	*N*	Keyword		
			1	2	3
Chinese Academy of Sciences	China	2322	Unclassified Drug	Drug Isolation	Drug Structure
Chinese Academy of Medical Sciences	China	1432	Chemistry	Unclassified Drug	Isolation And Purification
Peking Union Medical College Hospital	China	1200	Chemistry	Unclassified Drug	Isolation And Purification
Ministry of Education China	China	1192	Unclassified Drug	Controlled Study	Chemistry
China Pharmaceutical University	China	851	Unclassified Drug	Chemistry	Plant Extract
Kunming Institute of Botany Chinese Academy of Sciences	China	694	Unclassified Drug	Drug Isolation	Drug Structure
China Academy of Chinese Medical Sciences	China	650	Unclassified Drug	Chemistry	Drugs, Chinese Herbal
Universidade de Sao Paulo—USP	Brazil	626	Unclassified Drug	Plant Extract	Controlled Study
Shenyang Pharmaceutical University	China	598	Unclassified Drug	Chemistry	Drug Isolation
University of Chinese Academy of Sciences	China	549	Unclassified Drug	Controlled Study	Drug Isolation
UNESP-Universidade Estadual Paulista	Brazil	534	Unclassified Drug	Plant Extract	Controlled Study
Kyung Hee University	South Korea	533	Unclassified Drug	Controlled Study	Plant Extract
King Saud University	Saudi Arabia	533	Unclassified Drug	Plant Extract	Controlled Study
Beijing University of Chinese Medicine	China	533	Chemistry	Drugs, Chinese Herbal	Herbaceous Agent
University of Karachi	Pakistan	520	Unclassified Drug	Plant Extract	Drug Isolation
Zhejiang University	China	497	Unclassified Drug	Chemistry	Controlled Study
Seoul National University	South Korea	496	Unclassified Drug	Controlled Study	Plant Extract
Tehran University of Medical Sciences	Iran	461	Unclassified Drug	Plant Extract	Controlled Study
Universidad Nacional Autónoma de México	Mexico	453	Unclassified Drug	Plant Extract	Controlled Study
Université de Yaoundé I	Cameroon	451	Unclassified Drug	Plant Extract	Controlled Study
Peking University	China	434	Unclassified Drug	Chemistry	Isolation And Purification
Second Military Medical University	China	425	Unclassified Drug	Plant Extract	Controlled Study
Universidade Federal do Rio de Janeiro	Brazil	414	Unclassified Drug	Plant Extract	Controlled Study
CNRS Centre National de la Recherche Scientifique	France	410	Unclassified Drug	Plant Extract	Controlled Study
Universiti Putra Malaysia	Malaysia	406	Unclassified Drug	Plant Extract	Controlled Study

**Table 2:** Main authors in medicinal plants.

	Author	Scopus Author ID	*N*	Affiliation, Country	h-Index
1	Van Staden, J.	7201832631	238	University of KwaZulu-Natal, South Africa	69
2	Rahmatullah, M.	6701489271	175	University of Dhaka, Bangladesh	38
3	Huang, L.Q.	56156528000	150	China Academy of Chinese Medical Sciences, China	36
4	Choudhary, M.I.	35228815600	142	University of Karachi, Pakistan	53
5	Afolayan, A.J.	7003478648	137	University of Fort Hare, South Africa	41
6	Heinrich, M.	16156235300	124	UCL, London, United Kingdom	54
7	Khan, I.A.	26643155300	124	University of Mississippi, United States	54
8	Efferth, T.	7005243974	122	Johannes Gutenberg Universität Mainz, Germany	70
9	Farnsworth, N.R.	35392089500	118	University of Illinois at Chicago, United States	63
10	Rafieian-Kopaei, M.	6506929448	115	Shahrekord University of Medical Sciences, Iran	60
11	Kuete, V.	15757756200	114	University of Dschang, Cameroon	38
12	Xiao, P.G.	7103088959	113	Ministry of Education China, China	37
13	Vilegas, W.	7004140097	107	UNESP-Universidade Estadual Paulista, Brazil	36
14	Hao, X.J.	7202000647	105	Chinese Academy of Sciences, China	38
15	Sun, H.D.	7404828012	105	Kunming Institute of Botany Chinese Academy of Sciences, China	47
16	Li, P.	56381767900	101	China Pharmaceutical University, China	51

**Table 3:** Main keywords by country.

Rank	Country	*N*	1	2	3	4
1	China	19,846	Unclassified Drug	Chemistry	Controlled Study	Plant Extract
2	India	16,372	Unclassified Drug	Plant Extract	Controlled Study	Animal Experiment
3	USA	7339	Unclassified Drug	Plant Extract	Controlled Study	Chemistry
4	Brazil	5993	Unclassified Drug	Plant Extract	Controlled Study	Animal Experiment
5	Japan	4557	Unclassified Drug	Plant Extract	Drug Isolation	Controlled Study
6	South Korea	4131	Unclassified Drug	Controlled Study	Plant Extract	Animals
7	Germany	3867	Unclassified Drug	Plant Extract	Controlled Study	Chemistry
8	Iran	3771	Unclassified Drug	Plant Extract	Controlled Study	Essential Oil
9	United Kingdom	2377	Unclassified Drug	Plant Extract	Controlled Study	Chemistry
10	Pakistan	2220	Unclassified Drug	Plant Extract	Controlled Study	Chemistry
11	Italy	2135	Unclassified Drug	Plant Extract	Controlled Study	Chemistry
12	France	2031	Unclassified Drug	Plant Extract	Controlled Study	Drug Isolation

**Table 4:** Main keywords related to plant parts and plant families studied.

Part of the Plant	Documents	Main Family Studied	Keyword	*N*
Leaf-Leaves	14652	Asteraceae, Fabaceae, Lamiaceae	Plant Leaf	12,009
			Plant Leaves	4664
Root-Roots	9581	Asteraceae, Fabaceae	Plant Root	7695
			Plant Roots	3920
Seed	5204	Fabaceae, Asteraceae	Plant Seed	3789
			Seeds	2149
Stem	4480	Fabaceae, Asteraceae, Apocynaceae	Plant Stem	3561
			Plant Stems	1462
Fruit	4357	Fabaceae, Asteraceae,	Fruit	3423
			Fruits	259
Bark	3358	Fabaceae, Meliaceae, Euphorbiaceae, Apocynaceae, Asteraceae	Bark	3146
			Plant Bark	1171
Flower	2615	Asteraceae, Lamiaceae, Fabaceae	Flower	2081
			Flowers	804
Rhizome	2519	Zingiberaceae, Asteraceae	Rhizome	1969

**Table 5:** Plant families and Institutions.

Rank	Plant Family	Documents	Main Country	Main Affiliation (Country)
1	Fabaceae	4492	USA	Universidade de Sao Paulo – USP (Brazil)
2	Leguminosae	3255	USA	Wageningen University and Research Centre (Netherlands)
3	Asteraceae	2743	China	Chinese Academy of Sciences (China)
4	Lamiaceae	1825	China	Chinese Academy of Sciences (China)
5	Apocynaceae	962	India	Chinese Academy of Sciences (China)
6	Angiosperm	914	China	Chinese Academy of Medical Sciences & Peking Union Medical College (China)
7	Euphorbiaceae	898	India	Chinese Academy of Sciences (China)
8	Apiaceae (Umbelliferae)	884(135)	China	Tehran University of Medical Sciences (Iran)
9	Rubiaceae	814	India	Chinese Academy of Sciences (China)
10	Rutaceae	732	India	CNRS Centre National de la Recherche Scientifique (France)
11	Solanaceae	539	India	University of Development Alternative (Bangladesh)
12	Rosaceae	582	China	Chinese Academy of Sciences (China)
13	Compositae	352	China	Lanzhou University (China)

**Table 6:** Main keywords used by the communities detected in the topic in the period 2009–2014.

Cluster	Color	Main Keywords	Topic
1-1	Red	Human, Phytotherapy, herbaceous agent, traditional medicine, ethnobotany, diabetes mellitus	Traditional medicine
1-2	Green	Drug isolation, drug structure, chemistry, drug determination, molecular structure	Drug determination
1-3	Purple	Animal, mouse, mice, animal cell, apoptosis, anti-inflammatory effect, protein expression	Animals-in vivo study
1-4	Yellow	Unclassified drug, drug screening, flavonoid, phytochemistry, plant leaf	Unclassified drug
1-5	Blue	Drug efficacy, animal experiment, dose response, oxidative stress, histopathology	Drug efficacy
1-6	Cian	Solvent, ethanol, neuroprotection, acetic acid, sodium chloride	Effect of extraction solvent
1-7	Orange	antimalarial activity, antimalarials, *Plasmodium berghei*, *Plasmodium falciparum*	Malaria

**Table 7:** Main keywords used by the communities detected in the topic in the period 2015–2019.

Cluster	Color	Main Keywords	Topic
2-1	Red	Unclassified drug, chemistry, plant extract, phytochemistry, flavonoid	Unclassified drug
2-2	Green	Traditional medicine, herbaceous agent, phytotherapy, ethnopharmacology, drug efficacy	Traditional medicine
2-3	Blue	In vitro study, human cell, antineoplastic agent, cytotoxicity, apoptosis	Cancer
2-4	Cyan	In vivo study, male, oxidative stress, animal tissue, rat, antidiabetic activity, liver protection	In vivo study- antidiabetic activity
2-5	Purple	Metabolism, animal, anti-inflammatory activity, mouse, dose response	Animals- Anti-inflammatory activity

**Table 8:** Countries collaboration in the period 2009–2019.

Cluster	Color	Main Countries	Number of Countries	Leader
1	Green	Brazil, Italy, Turkey, Spain	16	Brazil
2	Grey	South Africa, Belgium, France, Morocco	14	South Africa
3	Blue	India, Iran, Iraq, Chile	12	India
4	Yellow	Germany, Pakistan, Saudi Arabia, Egypt	12	Pakistan
5	Purple	Indonesia, Malaysia, Thailand, Australia	10	Indonesia
6	Cian	USA, UK, Japan, Canada, South Korea	8	USA
7	Orange	Cameroon, Kenya, Denmark, Nepal	5	Cameroon
8	Red	China, Taiwan, Singapore, Hong Kong	5	China
